# Detection of Mitochondrial Mutations Through Isothermal Nucleic Acid Amplification Coupled With Clustered Regularly Interspaced Short Palindromic Repeat-Associated Endonuclease Cas13a

**DOI:** 10.3389/fgene.2020.622671

**Published:** 2021-01-12

**Authors:** Hua Jiang, Kun Duan, Xu Han, Jun Wang, Xiao Liu, Maoxiao Yan, Yunxiu Wang, Hongyan Liu, Huiling Shi, Xiaoqing Gao, Chuan Ouyang, Xue Fu, Xinxin Zhang, Chao Liu

**Affiliations:** ^1^Department of Otolaryngology, The Second Affiliated Hospital, Zhejiang University School of Medicine, Hangzhou, China; ^2^Hangzhou Matridx Biotechnology Co., Ltd., Hangzhou, China

**Keywords:** isothermal reaction, transcription-mediated amplification, clustered regularly interspaced short palindromic repeat, mitochondrial mutation, nonsyndromic hearing loss

## Abstract

The clustered regularly interspaced short palindromic repeat (CRISPR)-associated endonuclease Cas13a can specifically bind and cleave RNA. After nucleic acid pre-amplification, bacterial Cas13a has been used to detect genetic mutations. In our study, using a transcription-mediated amplification together with Cas13a, we can isothermally amplify and detect mitochondrial point mutations under non-denaturing conditions from human genomic DNA. Unlike previous reports, we prepared CRISPR DNA with T7 promoter sequences and generated CRISPR RNA *via* transcription-mediated amplification instead of synthesizing and adding CRISPR RNA in a separate step. As a proof-of-concept, we showed that both m.1494C > T and m.1555A > G mutations were detected within 90 min. In addition, we explored various designs of CRISPR DNA to improve assay specificity, including the location and number of nucleotide mismatches, length of protospacer sequence, and different buffering conditions. We also confirmed the possibility of a “one-step single-tube” reaction for mutation detection. This assay can robustly distinguish circular DNA templates that differ by a single nucleotide. It has the potential to be adapted for automated applications, such as the screening of mitochondrial diseases.

## Introduction

Since the invention of polymerase chain reaction (PCR) in the 1980s, nucleic acid test (NAT) has shown vast potential in the field of *in vitro* diagnostics. NAT enables the detection of genetic mutations and the clinical diagnosis of inherited diseases. Due to the limited amount of genetic materials in biological samples, NAT typically requires a nucleic acid amplification step, such as PCR (Mullis et al., 1986). PCR exponentially amplifies DNA through thermal cycling and is regularly performed in biomedical laboratories.

**Figure 1:**
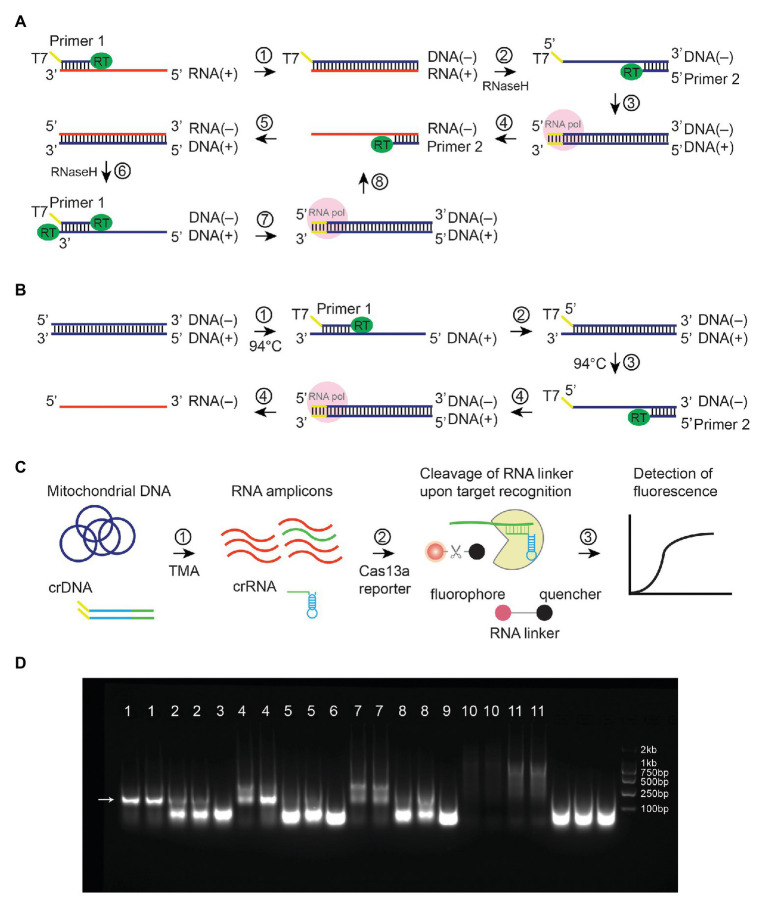
Illustrations of Transcription-Mediated Amplification. Mechanisms were shown for **(A)** TMA amplification of RNA. DNA strands were shown in blue, whereas RNA strands were in red. Plus DNA strand serves as a template for T7-mediated transcription that results in minus-strand RNA. **(B)** TMA amplification of linear dsDNA. A denaturing step (step 3) at 94°C was needed to allow the binding of primer 2. **(C)** TMA coupled with CRISPR-Cas13a to detect mitochondrial mutations. **(D)** TMA amplified plasmid at 41°C without denaturation steps. Lane 1, 10-ng template, 0.4-μM primers; lane 2, 1-ng template, 0.4-μM primers; lane 3, no template, 0.4-μM primers; lane 4, 10-ng template, 0.2-μM primers; lane 5, 1-ng template, 0.2-μM primers; lane 6, no template, 0.2-μM primers; lane 7, 10-ng template, 0.1-μM primers, lane 8, 1-ng template, 0.1-μM primers; lane 9, no template, 0.1-μM primers; lane 10, 1-ng template, no primers; lane 11, 10-ng template, no primers. An arrow showed expected amplicons.

Despite its widespread use, PCR has several issues when performed in a clinical setting. First, it requires a thermal cycler. Second, the experimental procedures involve multiple liquid handling steps and require well-trained personnel. Third, DNA aerosol is a potential source of cross-contamination and difficult to eliminate. In contrast, isothermal techniques such as transcription-mediated amplification (TMA) alleviate the need for a thermal cycler and could be superior to PCR in terms of simplicity and shorter turnaround time. TMA is used to amplify RNA (100–1,000 amplicons per cycle) at 40–42°C with the help of two enzymes: reverse transcriptase and RNA polymerase (Figure 1A; Stary et al., 1998). In contrast to PCR, amplified RNA molecules are less likely to cause contamination, as they are not stable (Aslanzadeh, 2004). When used in conjunction with a reporting assay (i.e., molecular beacon, hybridization protection assay, etc.), TMA enables pathogen detection and diagnosis of infectious diseases, especially RNA viruses (Swenson et al., 2016). However, TMA is generally not used to amplify double-stranded DNA (dsDNA) unless denaturing (>90°C) and annealing steps are added in each cycle to allow primer binding (Figure 1B).

Voisset et al. (2000) reported in their study that an assay named nucleic acid sequence-based amplification (NASBA) could amplify plasmid under non-denaturing conditions. NASBA is almost equivalent to TMA except for the choice of reverse transcriptase (NASBA uses AMV, whereas TMA uses M-MLV; Sahoo et al., 2013). The mechanism for NASBA to amplify plasmid DNA without denaturing remains obscure. We postulate that even at 40–42°C, circular dsDNA may partially unwind, allowing primer binding to occur. The observation that NASBA can amplify plasmid under non-denaturing conditions prompted us to hypothesize that such techniques can also isothermally amplify mitochondrial DNA, which is also circular dsDNA (Anderson et al., 1981). Because TMA and NASBA generate RNA amplicons, they could be followed by a reporting assay that detects RNA. For instance, the CRISPR-associated endonuclease Cas13a has been shown to promiscuously cleave RNA upon CRISPR RNA (crRNA)-guided binding of an RNA template (Gootenberg et al., 2017). Moreover, crRNA used in the Cas13a assay can be generated *via* T7-transcription in the TMA reaction, instead of synthesizing crRNA separately (Figure 1C).

As a proof-of-concept, we combined TMA with Cas13a and used it to detect mitochondrial mutations. The assay was performed isothermally under non-denaturing conditions (41°C) in a single tube. We named it CRISPR-mediated nucleic acid detection (CNAD). Using both plasmid and genomic DNA (gDNA), we showed that CNAD could detect target sequences or point mutations in <90 min, making it a faster and more convenient alternative to PCR.

## Materials and Methods

### Sample Collection

gDNA was acquired from patients admitted to the second affiliated hospital, Zhejiang University School of Medicine. QIAamp DNA Blood Mini Kit (Qiagen, 51,104) was used to extract gDNA (contains both chromosomal and mitochondrial DNA) from 200 μl of peripheral blood. The study design was approved by the Institutional Review Board at the second affiliated hospital, Zhejiang University School of Medicine (# 2013-011).

### Expression and Purification of LwaCas13a

A codon-optimized sequence encoding LwaCas13a was synthesized (Genscript) and cloned into a pET3a vector. After transformation [BL21(DE3) cells], a single colony was cultured overnight in 5 ml of Lysogeny broth medium. The resulting culture was diluted 100-fold and inoculated in 1 L of Lysogeny broth medium, which was incubated at 170 rpm, 37°C until an optical density at a wavelength of 600 nm reached 0.6. Isopropyl β-d-1-thiogalactopyranoside was added to the culture (cooled to 16°C, final concentration 0.5 mM) to induce protein expression for 20 h. The solution was centrifuged at 4°C 8,000 × *g* for 10 min, and precipitates were collected and stored at −80°C. Protein purification was carried out at 4°C. Briefly, 50-ml Ni-Lysis buffer [20-mM Tris-HCl (pH 8.0), 300-mM NaCl, 10-mM imidazole, 0.5% NP-40, 1-mM phenylmethylsulfonyl fluoride (PMSF), and 0.5-mM dithiothreitol] and 5 μl of 10 mg/ml DNase I were added to the precipitates. After resuspension, the solution was sonicated for 15 min, centrifuged at 12,000 × *g* for 30 min, and the supernatant was filtered (0.45-μm film) and aliquoted into microcentrifuge tubes. The supernatant was then incubated overnight at 4°C on a column with Ni-NTA Beads, washed three times by Ni-wash buffer I [20-mM Tris-HCl (pH8.0), 300-mM NaCl, 10-mM imidazole, 1-mM PMSF, and 0.5-mM DTT] and eluted by 75 ml of 250-mM elution buffer. The protein solution was concentrated by centrifuging at 4,000 rpm for 15 min in a 50-KD Ultrafiltration tube (Millipore). Sephadex-G25 was used for desalting and Q buffer A [50-mM Tris-HCl (pH8.0), 0.1-mM PMSF, and 1-mM DTT] was used for buffer exchange.

### Preparation of Clustered Regularly Interspaced Short Palindromic Repeat DNA

We synthesized and cloned a universal sequence into pUC57 vector (Genscript): TAATACGACTCACTATAGGGGATTTAGACTACCCCAAAAACGAAGGGGACTAAAAC, which contained the T7 promoter (underlined) and the direct repeat sequences. We synthesized CRISPR DNA (crDNA) primers with the protospacer sequence at 5' end and 22 nt of the universal sequence at 3' end. The double-stranded crDNAs were prepared by PCR-amplifying pUC57 using both the T7 and crDNA primers. Amplicons were purified (Axygen AP-PCR-250) and stored at −20°C.

### Clustered Regularly Interspaced Short Palindromic Repeat-Mediated Nucleic Acid Detection

One microliter of genomic DNA (containing both chromosomal and mitochondrial DNA) or plasmid template (20 ng for gDNA, 1 ng for plasmid unless stated otherwise) was added to 24-μl reaction buffer to make a final reaction volume of 25 μl. The solution contained 40-mM Tris-HCl (pH 8.0), 16-mM MgCl_2_, 40-mM KCl, 5-mM DTT, 8% (v/v) DMSO, 5-mM Sorbitol, 1 mM of each dNTP, 2 mM of each NTP, 0.2 μM of each primer, 10-U AMV (NEB, M0277L), 0.5-U RNase H (NEB, M0297L), 50-U T7 RNA Polymerase (NEB, M0251L), 20-U RNase inhibitor (NEB, M0314L), and 2.5-μg BSA. The mix was heated to 41°C for 60 min to allow amplification of the target sequence. The sequences of primers used for amplifying the *MTRNR1* gene were as follows (5'–3'): AATTCTAATACGACTCACTATAGGGAGAatttagcagtaaactaagagta (forward, T7 sequences are capitalized) and actctggttcgtccaagtgca (reverse).

**Figure 2:**
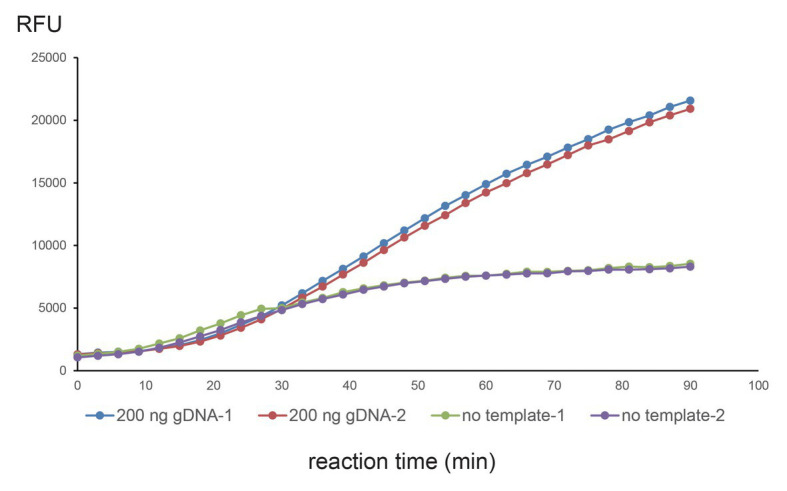
Single-tube one-step CNAD reaction using 200-ng gDNA. WT crDNA with two additional mismatches at seventh and ninth positions was used to detect WT mitochondrial DNA. Two hundred nanograms of genomic DNA or no template control was tested and repeated twice. Fluorescence (RFU) vs. reaction time was plotted. Each dot represents a fluorescence reading.

Amplification products were then diluted by reaction buffer (1:9 v/v). The final solution contained 40-mM Tris-HCl (pH 7.3), 8-mM MgCl_2_, 60-mM NaCl, 10-mM KCl, 10-mM DTT, 5% (v/v) PEG 4000, 0.5-μg purified LwaCas13a, and 160-nM FAM-RNase Alert (IDT). Emission fluorescence was excited by 490-nm blue light and recorded in a microplate reader at 520 nm at 37°C (TECAN, infinite M plex). For detection of m.1555 mutation, the crDNA sequence was cgcatttatatagaggagGGaTgtcgtagttttagtccccttcgtttttg (mismatches are capitalized). For one-step reaction in Figure 2, the crDNA sequence was gtacacaccgcccgtcacCGtGctcaaggttttagtccccttcgtttttg (mismatches are capitalized).

## Results

### Transcription-Mediated Amplification of Plasmid Under Non-denaturing Conditions

Transcription-mediated amplification (TMA) is an isothermal method for the amplification of RNA at 40–42°C (Figure 1A; Stary et al., 1998). It can also be adapted for amplification of dsDNA by incorporating denaturing and annealing steps (Figure 1B; Kamisango et al., 1999). Through a literature search, we found that an assay similar to TMA has been shown previously to amplify plasmid under non-denaturing conditions (Voisset et al., 2000). Indeed, we confirmed in our study that TMA could also amplify plasmid at 41°C without any denaturation steps (Figure 1D, amplicon size 185 bp). This phenomenon pointed to the potential use of TMA to isothermally amplify circular dsDNA templates.

### Transcription-Mediated Amplification Can Isothermally Amplify Mitochondrial DNA

We hypothesized that TMA could isothermally amplify mitochondrial DNA, which is circular dsDNA. To that end, we designed primers targeting the mitochondrial *MTRNR1* gene and tested the TMA reaction with/without primers using 20-ng gDNA. The resulting nucleic acids were purified and sequenced on an Illumina Nextseq platform (SE75). All reads were mapped to a reference genome (GRCh38.p13), and we compared the number of reads that were specific to *MTRNR1* and nuclear genes (Table 1). TMA resulted in targeted amplification of *MTRNR1* as shown by the *MTRNR1*/Chromosomal reads ratio (1.942 vs. 0.003%, experiment vs. no primer control, respectively). This experiment demonstrated that under non-denaturing conditions, TMA could amplify mitochondrial DNA.

**Table 1:** TMA amplification of mitochondrial gene from genomic DNA.

Sample	MTRNR1 reads	Mitochondrial reads	Chromosomal reads	MTRNR1/Chromosomal reads ratio
20-ng gDNA with primers	4,850	6,824	249,734	1.942%
20-ng gDNA without primers	26	7,859	941,768	0.003%

### Transcription-Mediated Amplification and Clustered Regularly Interspaced Short Palindromic Repeat-Associated Endonuclease Cas13a for Detecting Mitochondrial Mutations

After the TMA reaction, we used the RNA-binding endonuclease Cas13a as a reporting assay. The rationales are as follows: (1) TMA generates RNA amplicons, which are substrates for Cas13a (Shmakov et al., 2015) and (2) Target recognition is directed by crRNA, which can be generated *via* T7-mediated *in vitro* transcription. As a result, unlike previous studies, crRNAs do not have to be separately prepared. Instead, we could make crRNA by adding crDNA (with T7 promoter sequences) in the TMA reaction (Figure 1C). DNA is more stable than RNA and easier to prepare and store.

**Figure 3:**
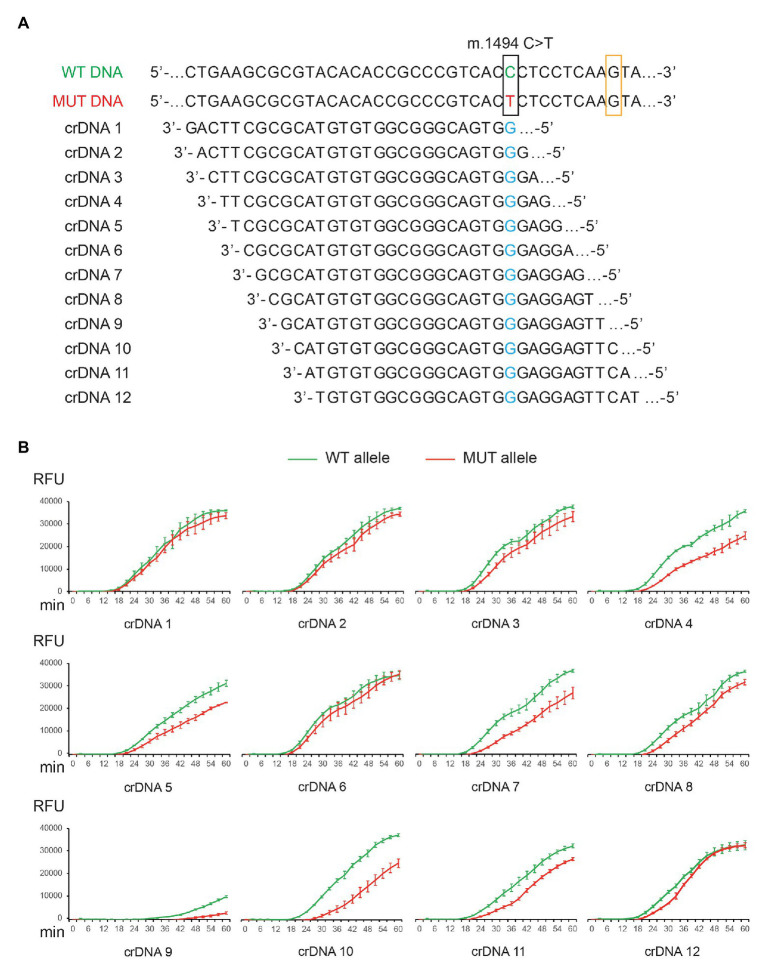
Detection of m.1494C > T mutation by CNAD. **(A)** Nucleotide sequences of the wild-type (WT) and mutant (MUT) mitochondrial DNA and 12 different designs of crDNAs were shown. **(B)** Each crDNA was used to detect plasmid containing either the WT or the MUT target sequence. Resulting fluorescence vs. reaction time was plotted. Each data point consists of two replicates. Error bars indicate SEM.

Next, we examined whether TMA + Cas13a (CNAD) can: (1) detect target sequence and (2) identify mitochondrial mutations. To that end, we tested CNAD in detecting m.1494C > T, which is a mitochondrial mutation that causes aminoglycoside-induced hearing loss (Zhao et al., 2004). First, we prepared plasmids containing either the wild-type (WT) or mutant (MUT) sequences. Then, we designed crDNAs in which the protospacer sequence (for target binding) fully matched the WT DNA (WT crDNAs). Therefore, for MUT DNA, the crDNA has a nucleotide mismatch (Figure 3A, marked blue). To determine whether the position of mismatch affects target binding and Cas13a activity, we tried placing the mismatch at positions 1 through 12 in the protospacer (the first 5'-nucleotide of protospacer is designated position 1, Figure 3A). Among all crDNAs tested, we found 4, 5, 7, 8, 9, 10, and 11 produced clear differences in fluorescence towards the WT and MUT sequences (Figure 3B, green vs. red). Notably, bacterial Cas13a has been shown to prefer H (A, U, or C) as the protospacer flanking site (PFS) for efficient targeting (the 3'-end nucleotide on the template that is adjacent to the protospacer sequence; Abudayyeh et al., 2016). For crDNA 9, the PFS happened to be guanine (G; Figure 3A, orange box), thus drastically weakening the overall signal (Figure 3B).

### Clustered Regularly Interspaced Short Palindromic Repeat DNA Design for Enhanced Specificity

The specificity of CRISPR-Cas13a in detecting point mutations depends on the binding affinity between crRNA and target sequences. The results obtained from 12 crDNAs indicated the importance of nucleotide mismatch in the protospacer. To further improve specificity, we chose crDNA 10 and tested the effect of introducing additional mismatches. This crDNA was selected among others, as it generated the most significant differences between on-target and off-target signal (Figure 3B, green vs. red).

**Figure 4:**
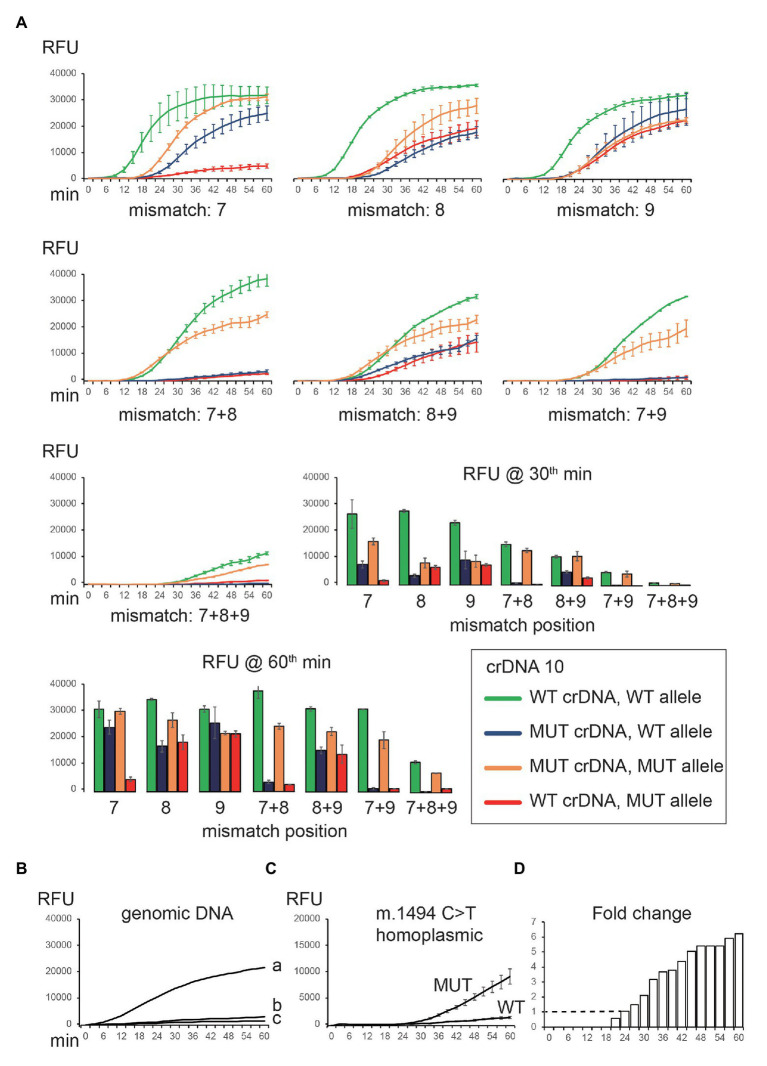
Optimization of crDNA design to detect mitochondrial mutation. **(A)** Additional nucleotide mismatches were introduced in the spacer of crDNA 10. Both WT and MUT crDNAs were tested against on-target (WT crDNA and WT plasmid, MUT crDNA and MUT plasmid) and off-target (WT crDNA and MUT plasmid, MUT crDNA and WT plasmid) templates, respectively. The resulting fluorescence was recorded and plotted. **(B)** CNAD detection of WT gDNA (a, 20 ng; b, 2 ng; and **c**, 0 ng) using WT crDNA. **(C)** CNAD detection of 20-ng gDNA containing homoplasmic m.1494C > T mutation. crDNA with mismatches at positions 7 and 9 was used. **(D)** Fold change of fluorescence generated from WT and MUT crDNA was shown. Each data point consists of two replicates. Error bars indicate SEM.

crDNAs 7, 8, 9, 10, and 11 were able to differentiate between WT and MUT sequences. We speculated that these mismatch positions might be critical for target binding. We tested this hypothesis by incorporating extra mismatches in crDNA 10, starting from position 7. The mismatches were prepared in a way that purines were changed to pyrimidines and vice versa (i.e., C–G, and A–T). In addition, we also examined the effect of double and triple mismatches. In total, we tested seven variations of crDNA 10 (mismatches at positions 7, 8, 9, 7 + 8, 8 + 9, 7 + 9, and 7 + 8 + 9; Figure 4A). The specificity improved further with more mismatches. However, signal intensity was also reduced (Figure 4A). We achieved optimal fluorescent intensity and on-/off-target signal ratio by using crDNAs with two mismatches (Figure 4A).

### Clustered Regularly Interspaced Short Palindromic Repeat-Mediated Nucleic Acid Detection of Mitochondrial Mutations From Genomic DNA

We tested crDNA with two mismatches (7 + 9) in identifying m.1494C > T from gDNA. TMA reagents, crDNA, and gDNA template were mixed in a reaction tube to allow amplification for 60 min. Then, Cas13a protein and reporters were added, and fluorescence was monitored. We were able to detect the signal using 20 ng (Figure 4B, a) but not 2 ng (Figure 4B, b) of WT gDNA. Next, we tested 20 ng of patient gDNA containing homoplasmic m.1494C > T mutation. As expected, only MUT, but not WT crDNA, yielded a signal (Figure 4C). If using fold change of two (on-/off-target fluorescence) as a reporting threshold, the mutation was detected at 24th min, making the sample results time <90 min (Figure 4D).

### Strategies for Optimizing Mutation Detection by Clustered Regularly Interspaced Short Palindromic Repeat-Mediated Nucleic Acid Detection

**Figure 5:**
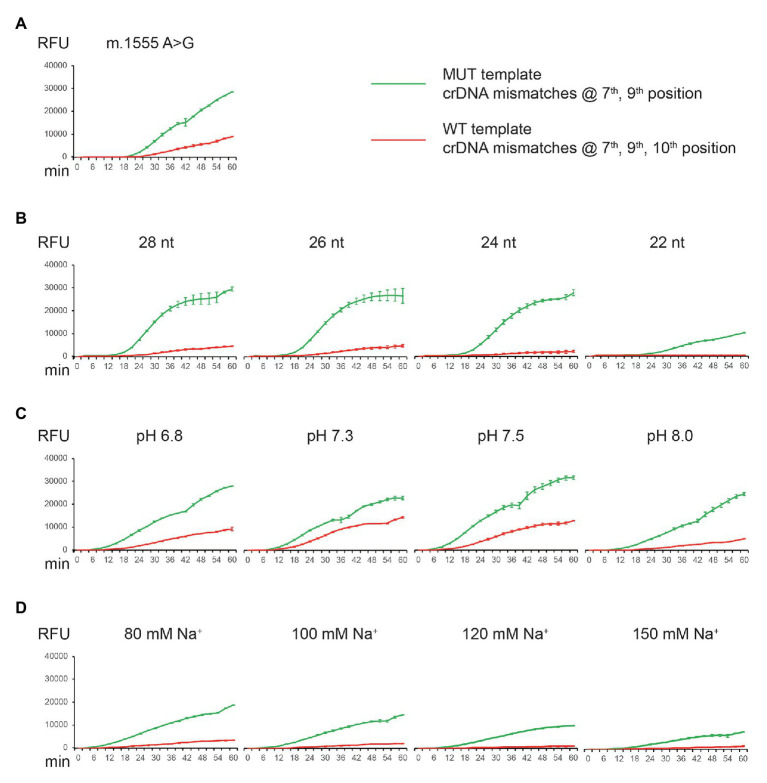
Detection of m.1555A > G mutation by CNAD and assay optimization. **(A)** MUT crDNA with mismatches at positions 7 and 9 was tested using both the MUT (on-target) and WT (off-target) plasmid templates. **(B)** Same crDNA was tested by altering the length of spacer sequence. **(C)** Same crDNA was tested at different pH. **(D)** Same crDNA was tested at different sodium concentrations. Each data point consists of two replicates. Error bars indicate SEM.

We explored different ways to improve the specificity of CNAD without severely reducing signal intensity. The approaches we found to be effective were: (1) change the position of nucleotide mismatch in the protospacer of crDNA; (2) introduce additional mismatches; (3) shorten the length of protospacer sequence; and (4) adjust the buffering conditions such as pH and sodium level. We have shown that m.1494C > T can be identified using the first two approaches (Figures 3B, 4A). However, when applying the same methods to detect another mitochondrial mutation [m.1555A > G, a mutation associated with aminoglycoside-induced nonsyndromic sensorineural deafness (Estivill et al., 1998)], the results varied. With three mismatches in the protospacer, the crDNA still produced an off-target signal (Figure 5A, red line). Therefore, we attempted other tactics to further improve specificity. First, we shortened the protospacer length. Both on- and off-target signal decreased as the protospacer length decreased, confirming that a shorter protospacer leads to higher specificity. However, the overall signal was drastically reduced using crDNA with a spacer length of 22 nt (Figure 5B). Then, we modified the pH and sodium concentrations and found that higher pH or sodium levels also improved specificity, albeit to a lesser extent (Figures 5C,D).

### Isothermal One-Step Reaction in a Single Tube

CNAD can isothermally amplify and detect point mutations in circular dsDNA. The assay was carried out in a single tube in two separate steps. Frist, TMA reagents, cDNAs, and template were mixed to produce target RNA amplicons and crRNAs; Second, Cas13a protein and reporters were added. The reaction tube was then placed into a microplate reader to record fluorescence.

We wondered whether the two separate steps could be merged as one, which would further reduce hands-on time and cross-contamination. We used WT crDNA and 200-ng gDNA and tested them in a one-step reaction (reagents, crDNA, template, Cas13a, and reporters were mixed at once). We were able to observe a clear signal (repeated twice). However, in the no-template control, a background signal was also seen (Figure 2). The on-target signal was apparently stronger, raising the possibility of one-tube, one-step CNAD for detecting mitochondrial mutations.

## Discussion

### Mitochondrial Mutations Are Associated With Nonsyndromic Hearing Loss

Hearing impairment affects approximately 1 in 1,000 newborns worldwide (Venkatesh et al., 2015). Nonsyndromic hearing loss is an important cause for hereditary hearing loss, which has several different patterns of inheritance, in which mitochondrial mutations including m.1494C > T and m.1555A > G in the *MTRNR1* gene are maternally inherited. The primary causes of maternally inherited sensorineural hearing loss are m.1555A > G and m.3243A > G mutations. Additional mutations such as m.1494C > T, m.7445A > G, 7472insC, and 7,511T > C were also reported and confirmed. The clinical features of mitochondrial hearing loss include (1) maternal inheritance; (2) hearing loss is sensorineural and primarily symmetrical; (3) variable penetrance and severity; and (4) childhood-onset (Mutai et al., 2017). Traditionally, mitochondrial mutations were detected by restriction fragment length polymorphism assay (Matsunaga et al., 2006), PCR and Sanger sequencing (Yao et al., 2019), or fluorescent PCR (Moraes et al., 2003).

### Pros and Cons of Clustered Regularly Interspaced Short Palindromic Repeat-Mediated Nucleic Acid Detection in Detecting Mitochondrial Mutations

CNAD can be performed in a single tube under isothermal conditions. The assay does not depend on expensive equipment and only requires portable and easily obtainable devices such as a water bath and fluorescence reader. This makes CNAD field-deployable, which can serve as a rapid mutation screening tool in developing regions of the world. In this study, we have shown that CNAD could detect plasmid or mitochondrial DNA templates that differed by a single nucleotide. The reaction time was within 90 min, including 60 min of TMA-mediated isothermal amplification and 30 min of Cas13a-mediated target recognition. Moreover, when TMA and Cas13a reagents were combined, the whole assay can be carried out in one step that results in a robust detectable signal after 30 min of reaction (Figure 2), suggesting that a faster sample-to-results time was achievable. In addition, as CNAD generates and detects RNA amplicons and is carried out in a single tube without the need to open caps throughout the experiment, it alleviates the problem of potential cross-contamination by DNA aerosols, which is one of the disadvantages of some PCR-based methods in mutation detection.

On the other hand, it is important to note that several PCR-based assays are also easy and fast to perform. The experiment would only require a simple setup and 40–50 min of reaction time. Moreover, qPCR-based methods can multiplex and detect more than one mutation in a single assay, which enables a more comprehensive screening of mitochondrial mutations and thus is advantageous to CNAD (currently a one-plex assay). In a previous report, CRISPR-associated proteins including Cas13a were shown to be capable of multiplexing and used to develop a four-channel assay for detection of four independent mutations in a single reaction (Gootenberg et al., 2018). It raises the possibility of multiplexing by CNAD *via* introducing additional Cas proteins into the reaction.

### Future Development and Ways to Improve Clustered Regularly Interspaced Short Palindromic Repeat-Mediated Nucleic Acid Detection

We have explored several ways to improve the specificity of CNAD without affecting the overall signal intensity. In our study, we found that an optimal specificity in differentiating templates that differ by a single nucleotide is achieved by introducing three synthetic nucleotide mismatches in the spacer sequence of crDNA. This finding was somewhat different from a previous study in which CRISPR-Cas13a was used to detect RNA virus, and one mismatch was introduced for recognizing single nucleotide polymorphism between four dengue virus serotypes, as well as region-specific strains of Zika viruses (Myhrvold et al., 2018). This discrepancy was probably due to a difference in target sequences. We also observed in our study that the crDNA design needs to be optimized for each different mutation, as illustrated by our effort to detect m.1555 mutation. The plasmid containing WT sequences elicited a significant background signal using crDNA with three nucleotide mismatches, which was not seen in the case of m.1494 mutation (Figure 4A). Therefore, a universal crDNA design strategy is needed for the assay to be more adaptable for detecting different mutations. Moreover, CNAD would greatly benefit from previously published techniques such as heating unextracted diagnostic samples to obliterate nucleases (Myhrvold et al., 2018), which enables an even faster and more convenient mutation detection assay without the need for nucleic acid extraction.

## Conclusion

We developed an assay utilizing TMA and CRISPR-Cas13a to isothermally amplify and detect targeted sequences in circular dsDNA templates, such as plasmid and mitochondria. This assay can be performed in one step in a single tube without the need for a thermal cycler. CNAD can robustly distinguish templates that differ by a single nucleotide and may be suitable for automatic mutation screenings.

## Data Availability Statement

The fastq files of the Next-Generation Sequencing experiment were deposited in the Sequence Read Archive (SRA) of NCBI and can be downloaded *via* the accession numbers SRR13149057 and SRR13149056.

## Ethics Statement

The studies involving human participants were reviewed and approved by the Institutional Review Board (IRB) at the second affiliated hospital, Zhejiang University School of Medicine (# 2013-011). The patients/participants provided their written informed consent to participate in this study.

## Author Contributions

HJ and CL conceived of this study and designed experiments. KD, XH, JW, HS, XG, CO, XF, and XZ developed the methods and performed experiments. XL, MY, YW, and HL carried out data analysis and contributed to the interpretation of results. All authors provided critical feedback and helped to shape the research. CL wrote the manuscript. All authors contributed to the article and approved the submitted version.

### Conflict of Interest

KD, JW, XH, HS, XG, CO, XF, XZ, and CL are employees at Hangzhou MatriDx Biotechnology Co., Ltd. The method described in this study has been filed for a patent application (CN201811001245.0).
